# The Influence of Invariant Natural Killer T Cells on Humoral Immunity to T-Dependent and -Independent Antigens

**DOI:** 10.3389/fimmu.2018.00305

**Published:** 2018-02-22

**Authors:** Mark L. Lang

**Affiliations:** ^1^Department of Microbiology and Immunology, University of Oklahoma Health Sciences Center, Oklahoma City, OK, United States

**Keywords:** CD1d, Natural Killer T, vaccine, humoral immunity, pathogen

## Abstract

Vaccination with CD1d-binding glycolipid adjuvants and co-administered protein, lipid, and carbohydrate antigens leads to invariant natural killer T (NKT) cell-dependent enhancement of protective B cell responses. NKT cell activation boosts the establishment of protein antigen-specific B cell memory and long-lived plasma cell (LLPC) compartments. NKT cells may exert a similar effect on some carbohydrate-specific B cells, but not lipid-specific B cells. The mechanisms of action of NKT cells on B cell responsiveness and subsequent differentiation into memory B cells and LLPC is dependent on CD1d expression by dendritic cells and B cells that can co-present glycolipids on CD1d and antigen-derived peptide on MHCII. CD1d/glycolipid-activated NKT cells are able to provide help to B cells in a manner dependent on cognate and non-cognate interactions. More recently, a glycolipid-expanded subset of IL-21-secreting NKT cells known as NKT follicular helper cells has been suggested to be a driver of NKT-enhanced humoral immunity. This review summarizes established and recent findings on how NKT cells impact humoral immunity and suggests possible areas of investigation that may allow the incorporation of NKT-activating agents into vaccine adjuvant platforms.

## Introduction

Several research groups have demonstrated that CD1d-restricted natural killer T (NKT) cells influence the humoral immune response to viruses, bacteria, their toxins, parasites, and fungi (Table 1). Typically prophylactic immunization of a mammal with a vaccine antigen or other pathogen product in combination with a CD1d-binding, NKT-activating adjuvant such as the α-galactosylceramide (α-GC) glycolipid has resulted in the enhancement of pathogen-specific Ab responses. These NKT-enhanced Ab responses are associated with, or contributory to enhanced protection against lethal challenges with pathogens or their toxins. The NKT-enhanced Ab responses are also typified by Ig class switch (1–4), establishment of B cell memory (Bmem) (2, 5), and long-lived plasma cells (LLPC) (6, 7), all hallmarks of a desirable vaccine response.

**Table 1:** List of pathogens and their products where immunization- or infection-induced natural killer T (NKT) activation influences protective humoral immunity.

Pathogen	Product/Antigen	Host species	Reference
Influenza PR8	Inactive PR8, live attenuated PR8, PR8 HA	Mouse	(8–10)
Influenza H3N2	Inactive H3N2	Mouse	(2)
Influenza	DNA vaccine (M2)	Mouse	(11)
Influenza H5N1	DNA vaccine (HA)	Mouse	(12)
Influenza (various)	HA	Mouse	(13)
Influenza H1N1	Inactivated or UV-killed H1N1	Pigs	(14–16)
Herpes simplex virus 1		Mouse	(17)
Herpes simplex virus 2	HSV-2 glycoprotein D (gD)	Mouse	(18)
Hepatitis B virus	HBsAg	Human, Mouse, Monkey (*Macaca fascicularis*)	(19)
Human herpes virus 8	None (blood samples following natural infection)	Human	(20)
Clade C HIV-1	Envelope gp140	Mouse	(21)
*Bacillus anthracis*	Anthrax toxin (AnTx)	Mouse	(3, 22, 23)
*Borrelia hermsii*	Live bacteria	Mouse	(24)
*Borrelia burgdorferi*^a^	Live bacteria	Mouse	(25, 26)
*Clostridium difficile*	Toxin B (TcdB)	Mouse	(27)
*Clostridium tetani*	Tetanus toxoid	Mouse	(2)
*Hemophilus influenzae*	P6 protein	Mouse	(28)
*Streptococcus pneumoniae*	Polysaccharide/liposomes, pneumococcal polysaccharide vaccine	Mouse	(29, 30)
*Plasmodium berghei*	Merozoite surface protein 1	Mouse	(31, 32)
*Toxoplasma gondii*	Live parasites	Mouse	(33)
*Trypanosoma cruzi*^a^	Live parasites	Mouse	(34–36)

*In response to the pathogens indicated by superscript “a,” some groups observed that NKT activation enhanced humoral immunity, while others reported that NKT cells were dispensable for the response*.

These findings support the notion that NKT cells could be harnessed following prophylactic vaccination to improve existing vaccines or contribute to the development of new vaccines. Arguably, to understand how best to harness NKT cells during vaccination, and/or how to appropriately direct a humoral immune response, the intersection of NKT cell and B cell biology needs to be understood. In this article, we discuss what is known about the mechanisms by which invariant NKT cells influence humoral immunity. We also discuss whether NKT-activating adjuvants can or should be incorporated into vaccines. Type II NKT cells expressing diverse TCRs (dNKT) are fully discussed elsewhere (37, 38), but briefly described herein in the context of vaccination.

## Mechanisms Regulating NKT Cell Influence on T-Dependent Humoral Immunity

**Figure 1:**
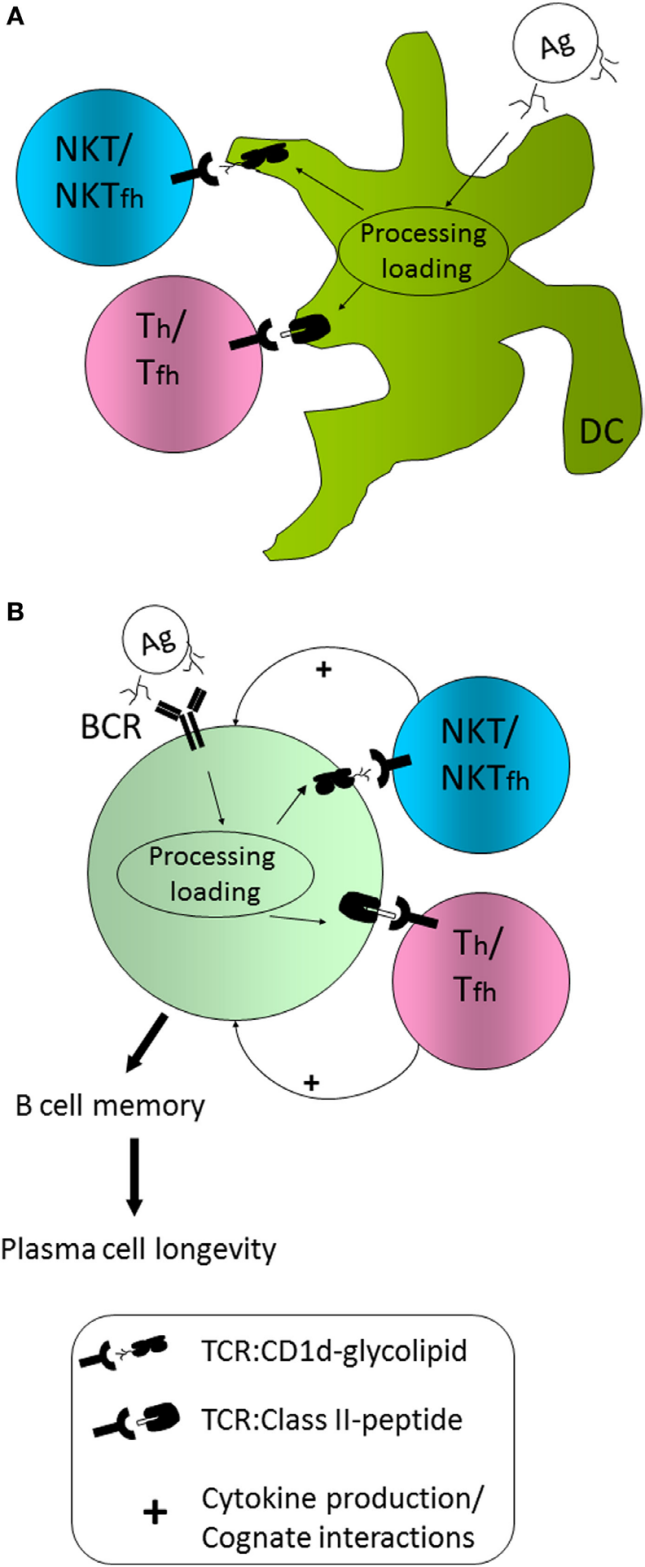
Model for natural killer T (NKT) cell influence on humoral immunity **(A)** CD1d^+/+^ dendritic cells (DCs) are able to capture, internalize, process, and present peptide Ag on MHCII and glycolipid Ag on CD1d and do so in a coordinated fashion. As a result, Th cell priming occurs, as does NKT activation and/or NKT follicular helper cell (NKTfh) differentiation. **(B)** B cells capture Ag *via* the BCR, but also capture complexed CD1d-binding glycolipid, or internalize it by endocytosis. B cells are, thus, able to coordinately present peptide on MHCII and glycolipid on CD1d. Consequently, B cells are able to receive help from DC primed or activated classical Th/Tfh cells as well as NKT/NKTfh cells. The additional help from NKT/NKTfh cells enhances the establishment of a Bmem compartment and the generation of long-lived plasma cells.

As mentioned, co-administration of a protein Ag and α-GC leads to enhanced humoral immunity against the protein Ag in a manner that is CD1d-dependent, and NKT cell-dependent (39). A model for how the humoral response is initiated is shown in Figure 1. In this model, professional APCs including classical CD11c^+^ dendritic cells (DCs) capture both the Ag and α-GC by endocytic mechanisms. This allows the internalization and trafficking of Ag and adjuvant (α-GC) into late endosomal processing compartments known as MIIC (MHC Class II compartments). It is in these compartments that protein-derived peptides and α-GC intersect with MHC II and CD1d, respectively (40, 41). Using well-defined mechanisms, peptide is loaded on MHCII and α-GC on CD1d [reviewed in Ref. (42, 43)]. The MHCII/peptide and CD1d/α-GC complexes are then transported to the cell surface for presentation to classical CD4^+^ T cells and NKT cells, respectively. Evidence also suggests that presentation of MHCII/peptide and CD1d/α-GC is facilitated by plasma membrane micro-domains or “rafts” (44, 45).

In the model (Figure 1A), Th priming by DCs is concordant with initial activation of NKT cells. In previous studies, our laboratory generated mixed bone marrow chimeric mice in which 50% of DCs expressed the diphtheria toxin receptor (DTR) under control of the CD11c promoter and the other 50% of cells were non-transgenic and CD1d^+/+^ or CD1d^-/-^ (46). Administration of DT temporarily ablated DTR transgenic CD1d^+/+^ DCs, leaving non-transgenic CD1d^+/+^ or CD1d^-/-^ DCs intact. In those experiments, Ab titers were similar between the groups. However, complete ablation of DTR^+^; CD1d^+/+^ DCs delayed the α-GC-enhanced Ab response, suggesting a contribution by CD1d^+/+^ DCs (46). Since that experiment, a Cre-Lox system has been employed by the Bendelac group to permanently ablate only CD1d^+/+^ DCs, showing a definitive contribution of these DCs to the humoral response to pneumococcal capsular polysaccharides (29). Although, a direct contribution of CD1d^+/+^ DCs to T-dependent humoral responses has not been formally demonstrated, it appears likely that they are required for NKT-enhanced responses.

In the model (Figure 1B), B cells specific for the immunizing Ag capture native Ag *via* the BCR and internalize α-GC by endocytosis, leading to MHCII and CD1d co-presentation by B cells. This will allow B cells to receive classical T cell help from Th cells and additional help from NKT cells. As a result of coordinated Th- and NKT-mediated B cell help, germinal center entry, Ig class switch, Bmem differentiation, and establishment of LLPC compartments are enhanced. Our laboratory performed adoptive transfers of CD1d^+/+^ and CD1d^−/−^ B cells into recipient μMT mice and demonstrated that B cell CD1d expression was essential for NKT-enhanced responses to the co-administered protein Ag (47). Co-presentation on MHCII and CD1d was further supported by Barral and colleagues who used liposomes containing Ag and α-GC for immunization (48).

These results raised the question of whether cognate interactions between B cells and NKT cells were occurring and dependent on CD1d and Vα14 TCR expression, respectively. In support of a direct B: NKT interaction and possible cognate interaction is our previous study adoptively transferring CD1d^+/+^ and CD1d^−/−^ B cells (47). Chang and colleagues used intra-vital microscopy to demonstrate direct interaction between HEL-specific MD4 B cells and NKT cells *in vivo* (49). The interactions lasted for 4–50 min suggesting a direct but time-limited interaction. The van den Elzen group showed that a combination of retinoic acid and α-GC led to reduced expression of CD1d by B cells, arguing for a constrained time window for B:NKT interaction (50). The Terhorst laboratory have also reported that signaling lymphocyte activation molecule associated protein (SAP) is expressed by NKT cells, but seems to be dispensable for initial B cells responses such as IgM production, but contributes to germinal center responses and, thus, class switch and somatic hyper-mutation (51). It should also be noted that Tonti and colleagues have observed cognate and non-cognate interactions between CD1d^+/+^ B cells and NKT cells (52). This suggests that the particular Ag, the dose and formulation (particulate versus soluble or linked versus separate Ag and adjuvant), and perhaps the route of immunization could influence the degree to which enhanced Ab responses rely on B cell CD1d expression. However, on balance, the evidence that CD1d^+/+^ B cells directly interact with NKT cells, and that this is required for NKT-enhanced humoral immunity is quite compelling.

Fewer studies have addressed whether there is direct communication between Th/Tfh and NKT/NKT follicular helper cells (NKTfh) cells during a humoral response. Our studies showed a temporal relationship between Th/Tfh and NKT/NKTfh production of IL-4 and IL-21, with the NKT/NKTfh compartments providing an early source of IL-21 (27). However, we did not detect any direct dependence of one cell type upon the other with regard to cytokine secretion.

While there is good evidence in support of a CD1d-dependent mechanism for B cell stimulation of NKT cells, it is somewhat less clear how the NKT provides help to the B cell. For example, using mixed bone marrow chimeras in which NKT cells were either CD40L^+/+^ or CD40L^−/−^, equal Ab responses to Ag and α-GC were observed (4). ICOS could not be studied in a similar manner because it is required for peripheral NKT survival (53), but *in vitro* assays suggested its requirement for NKT activation of marginal zone B cells (54). Given the propensity of marginal zone B cells to respond to T-independent Ags, its role in NKT-enhanced T-dependent responses remains unclear. It is difficult to envision CD40L and ICOS having no role to play in NKT-enhanced humoral responses, but experimental systems whereby these ligands are missing from the cell surface may be compensated by the same signals derived from Th cells. Alternatively, these co-receptor signals may be genuinely dispensable for NKT-mediated B cell help. If so, then the mechanisms of NKT- and Th-mediated B cell help are distinct.

Some evidence supports a role for NKT-derived soluble factors in B cell responses. The NKT cellular compartment is prolific in its rapid IFNγ and IL-4 section following α-GC activation, yet in the context of additional Th-mediated cytokine responses, NKT-derived cytokines may play a fairly limited role in influencing isotype switch. In bone marrow chimeras whereby NKT cells lacked IFNγ or IL-4, there were only modest effects on Ig class switch (3). A new study, however, reported that IL-4-secreting NKT cells positioned at the edge of the B cell follicle can promote germinal center entry, perhaps providing a mechanism of NKT-enhanced B cell memory (55). However, different laboratories have reported that α-GC leads to differentiation and expansion of a subset of NKT cells that display the hallmarks of T follicular helper cells (Tfh) and are, therefore, referred to as NKTfh cells (49, 56–58). This phenomenon explains the previous identification of an IL-21-secreting NKT subset (59), which is now known to express high levels of the master transcriptional regulator Bcl6, and upregulate the chemokine receptor CXCR5, and the PD1 molecule. The NKT subset may provide an early source of IL-21 (27) and perhaps accelerate Ig class switch, an effect that may have been missed in earlier studies examining cytokine contributions (3). The NKT-enhanced IgG response to T-dependent Ag is typically IgG1-dominated and this makes sense given the pivotal role of Tfh-derived IL-21 in IgG1 class switch (60, 61).

As mentioned, NKT activation is associated with increased numbers of LLPC (6, 7). Some mechanistic insights have been gained through bone marrow chimera experiments in which NKT cells lacked expression of either B cell activating factor (BAFF), a proliferation-inducing ligand (APRIL), or both BAFF and APRIL. While NKT-derived BAFF was dispensable for LLPC responses, APRIL made a modest contribution to longevity. However, the combination of BAFF and APRIL were critical for LLPC survival. In controls, bone marrow plasma cell numbers were maintained over around 90 days after immunization with minimal attrition. In the absence of NKT-derived BAFF and APRIL, there was a ~90% loss with 26 days (7). These data suggest a direct effect of NKT-derived plasma cell survival factors on the endurance of a humoral immune response.

## Mechanisms Regulating NKT Cell Influence on T-Independent Humoral Immunity

Studies by our group demonstrated that Abs complexed to a biotinylated α-GC could be used to stimulate BCR-dependent uptake, trafficking, loading, and presentation by CD1d (41). This Ag presentation pathway resulted in 100- to 1,000-fold more efficient activation of NKT hybridoma cells and suggested a hypothesis that such pathways could stimulate NKT-driven production of glycolipid-specific Abs. Indeed, the Brenner group demonstrated that anti-nitrophenol (NP) hapten Abs could be produced in a CD1d-/NKT-dependent manner following immunization with an NP-modified α-GC (62). The humoral response to NP-α-GC was examined and found to stimulate short-lived IgM responses without the establishment of Ab recall responses and B cell memory (62). In a further study, the B cell response to glycolipids was attributed to NKTfh cells (58). Therefore NKT (and NKTfh cells) cells may be able to boost Bmem responses to T-dependent Ags but not T-independent lipid Ags.

The Bendelac group, however, demonstrated a role of NKT/NKTfh cell-driven anti-polysaccharide responses (29). In a study involving immunization with capsular pneumococcal polysaccharides and α-GC, class-switch recombination, affinity maturation, and B cell memory were observed and there was a limited induction of NKTfh cell responses (29). In some unpublished studies from our laboratory, we have been unable to observe convincing Ab recall responses to T-independent carbohydrate Ags co-administered with α-GC, although there is a good adjuvant effect on primary responses (*Lang, unpublished observation*).

Clearly, information on the influence of NKT and NKTfh cells on humoral immunity to T-independent Ags is limited. More study is warranted in this area, particularly with regard to Ags associated with pathogenic bacteria.

## Considerations for Using NKT Cell-Activating Vaccines

The α-GC adjuvant has been valuable in helping delineate mechanisms of action by which NKT cells impact humoral immunity. However, several questions remain as to how best to move forward to incorporating NKT activation strategies into vaccines. The α-GC adjuvant is particularly potent *in vivo* and has the potential to initially activate all Type I NKT cells expressing the Vα14 TCR. There have been numerous reports detailing NKT cell anergy whereby a single treatment with α-GC can induce long-term NKT hypo-responsiveness to further stimulation (63–65). However, route of immunization may be contributory to this effect. Intradermal, subcutaneous, and mucosal vaccination routes allow repeat immunization and NKT responsiveness whereas intravenous and intraperitoneal delivery tends to result in anergy (6, 66–68). Some of the mechanisms underlying NKT anergy have been delineated and there are signaling pathways, such as CARMA1 and PD-1 that can be targeted to minimize anergy in mouse models (69, 70). While PD-1 blockade might be of practical value in cancer immunization, it is likely impractical for routine prophylactic vaccination in the field. A study in mice whereby α-GC was administered by the intra-tracheal route led to airway NKT cell activation and exacerbated airway hyper-reactivity and inflammation which is worth considering as a potential caveat to intranasal administration (71). These studies demonstrate that a combination of adjuvant selection, formulation, route of delivery, and perhaps mitigation of anergy-driving mechanisms may have to be considered when incorporating CD1d ligands into vaccines.

There are now several variants based on the α-GC molecule that can attenuate or enhance NKT activation [reviewed in Ref. (72)]. The α-GC molecule can be modified in its acyl chain, sphingosine chain, or sugar head-group and there is, therefore, considerable room for manipulating its effects on NKT cells. Furthermore, the Th1/Th17 to Th2 balance can be modulated by altering the α-GC molecule. Depending on the type of immune response that is desired, a different α-GC-derived adjuvant could be used for vaccination, perhaps with weaker anergy-inducing effects.

The vaccine formulation itself should be considered. Physically linking or associating vaccine antigens with α-GC (or a derivative thereof) is more likely to ensure that the same DCs and B cells that capture the vaccine, and coordinately present peptide on MHCII/HLA-2 and α-GC on CD1d. Small soluble complexes may result in different outcomes from larger (~100 nm) particles where extra-follicular B cell responses were observed in mice. Selection of the best particle size for ensuring that follicular and perhaps germinal center responses are worth considering.

Several studies have shown that α-GC is safe and well-tolerated when administered intravenously to cancer patients either in free form, or as part of a DC vaccine (73–77). It is, therefore, likely to be safe for inclusion in vaccines, but a few studies in mice implicated administration of α-GC during the third trimester in pregnancy loss, late preterm birth, and neonatal mortality (78–80). This issue, therefore, warrants additional attention to determine if α-GC adjuvants should be avoided in pregnancy.

This article has focused heavily on Type I invariant NKT cells. Type II NKT cells exhibit diverse TCR usage and respond to a growing list of CD1d-binding molecules. Available information on Type II NKT cells is reviewed elsewhere (37). We observed that the Th2 cytokine response to Alum adjuvant was attenuated by around 65% in mice lacking Type II NKT cells but not Type I NKT cells (81). This observation warrants further investigation and in the context of protection against pathogenic challenges. However, it should perhaps also be considered that Alum is safe, and stimulates excellent Th2 responses and poor Th1 responses. The inclusion of Type I NKT cell-activating adjuvants into vaccines containing Alum could potentially result in coordinated Type I and Type II NKT responses and give a broader response to existing vaccines.

## Concluding Remarks

The α-GC adjuvant has made possible valuable insights into how NKT cell and B cell biology intersect and provides a good jumping off point for the inclusion of similar adjuvants in vaccines. Potentially, derivatives of α-GC could be used to enhance, broaden, and extend the protective humoral response to a variety of protein and non-protein antigens.

## Author Contributions

The author confirms being the sole contributor of this work and approved it for publication.

## Conflict of Interest Statement

The author declares that the research was conducted in the absence of any commercial or financial relationships that could be construed as a potential conflict of interest.

## Abbreviations

Bmem, memory B cells; LLPC, long-lived plasma cells; NKTfh, NKT follicular helper cells; α-GC, alpha-galactosylceramide.
